# A Qualitative Study of Parental Decision‐Making Following a Prenatal Diagnosis of Turner Syndrome: “How Serious Is This?”

**DOI:** 10.1002/jgc4.70298

**Published:** 2026-10-09

**Authors:** Inger Lily Hjuler Dorf, Stina Lou, Christine Manich Bech Baggesgaard, Rune W. Naeraa, Casper P. Hagen, Anne Skakkebæk

**Affiliations:** ^1^ Department of Clinical Medicine Aarhus University Aarhus Denmark; ^2^ Department of Clinical Genetics Aarhus University Hospital Aarhus Denmark; ^3^ Department of Molecular Medicine Aarhus University Hospital Aarhus Denmark; ^4^ Department of Endocrinology Aarhus University Hospital Aarhus Denmark; ^5^ Center for Fetal Diagnostics Aarhus University Hospital Aarhus Denmark; ^6^ DEFACTUM – Public Health Research, Central Denmark Region Aarhus Denmark; ^7^ Department of Growth and Reproduction Copenhagen University Hospital – Rigshospitalet Copenhagen Denmark; ^8^ International Center for Research and Research Training in Endocrine Disruption of Male Reproduction and Child Health (EDMaRC) Copenhagen University Hospital – Rigshospitalet Copenhagen Denmark; ^9^ Department of Pediatric and Adolescent Medicine Aarhus University Hospital Aarhus Denmark; ^10^ Department of Clinical Medicine University of Copenhagen Copenhagen Denmark

**Keywords:** genetic counseling, parental decisions, prenatal diagnostics, qualitative research, termination of pregnancy, turner syndrome

## Abstract

A prenatal diagnosis of Turner syndrome (TS) presents expectant parents with complex and emotionally demanding decisions under conditions of uncertainty. However, there is limited insight into how parents interpret and integrate medical information, personal experiences, and values during this process. This qualitative study explores how parents who chose to continue a pregnancy following a prenatal TS diagnosis experienced and navigated decision‐making. The study was conducted within the Danish healthcare system between September 2024 and October 2025. During this period, we conducted 12 semi‐structured interviews with 19 parents of girls diagnosed prenatally with TS (seven joint interviews with both parents and five interviews with the mother only). Interviews were audio‐recorded, transcribed verbatim, and analyzed using reflexive thematic analysis. Across interviews, parents repeatedly returned to the question, “How serious is this?”, and our analysis showed that decision‐making unfolded as a dynamic, evolving process rather than a single point of choice. This process was captured through five interrelated themes: *The crisis of diagnosis*; *What parents bring into decision‐making*; *Contrasting images of Turner syndrome*; *What kind of life is possible?*; and *The turning point*. These themes illustrate how assessments of seriousness were shaped through an interplay of prior experiences, emerging attachment to the fetus, ultrasound findings, counseling encounters, and social responses. Over time, parents developed a more coherent understanding of the diagnosis, culminating in the decision to continue the pregnancy. In this way, decision‐making following a prenatal TS diagnosis extended beyond the evaluation of medical information and involved substantial interpretative and emotional work. Clinical encounters were most meaningful when information was contextualized and aligned with parents' concerns about everyday life and future possibilities. These findings reinforce the importance of prenatal counseling that integrates medical information with support for the interpretative and emotional work through which parents make sense of the diagnosis and navigate uncertainty.

## Abstract (highlights)

**What Is Known About the Topic:**

Parental decisions following a prenatal diagnosis of Turner syndrome have been associated with factors such as fetal karyotype, ultrasound findings, gestational age at diagnosis, and the framing of information in prenatal counseling. However, there is limited qualitative insight into *how* these elements shape parents' understanding of the diagnosis and are integrated into decision‐making.

**What This Paper Adds to the Topic:**

This study shows that the decision‐making process revolved around an ongoing effort to assess the perceived seriousness of the diagnosis in the context of parents' own lives. This understanding developed through an interplay of clinical encounters, prior experiences, and social context and shaped the decision to continue the pregnancy.

## Plain Language Summary

When parents learn during pregnancy that their baby has Turner syndrome, they may be confronted with decisions about whether to continue or terminate the pregnancy. This study shows how parents worked step by step to understand what the diagnosis would mean for their lives and for their child, using information from healthcare professionals, their own experiences, and support from others. This process led them to decide to continue the pregnancy and provided insight into how counseling can support decision‐making.

## 1 Introduction

Turner syndrome (TS) is a sex chromosome aneuploidy (SCA) caused by the complete or partial absence of one X chromosome and affects approximately 1 in 2000 live‐born girls (Gravholt et al. 2023, 2024; Berglund et al. 2020). TS is clinically heterogeneous, and this heterogeneity is also reflected in diverse lived experiences, with the significance of the condition varying across individuals and over the life course (Gravholt et al. 2023, 2024). As a condition involving variation in the number or structure of the X chromosome, TS can also be considered within the broader spectrum of sex chromosome variation (Griffiths 2018). Characteristic clinical features may include short stature, a higher prevalence of congenital malformations—particularly cardiac and renal—as well as gonadal dysgenesis, which often leads to premature ovarian insufficiency and infertility (Gravholt et al. 2023, 2024). The intelligence quotient is typically within the normal range, although specific learning difficulties are more prevalent than in the general population (Gravholt et al. 2023, 2024).

With the expansion of prenatal screening, TS is increasingly prenatally diagnosed, most often as an incidental finding through routine screening rather than targeted testing (Viuff et al. 2015). A prenatal genetic diagnosis is often experienced by parents as unexpected and emotionally disruptive, particularly when the condition is unfamiliar, confronting them with a complex and time‐sensitive decision about whether to continue or terminate the pregnancy (Hodgson et al. 2016; Damm et al. 2025; Pieters et al. 2011).

Research on parental decision‐making following a prenatal diagnosis of TS has primarily relied on quantitative or retrospective designs. In a recent systematic review, our research group synthesized findings from 27 studies examining factors associated with parental decisions to continue or terminate pregnancies affected by TS (Dorf et al. 2025). The decision to terminate the pregnancy was associated with factors such as ultrasound‐detected anomalies, fetal karyotype, gestational age at diagnosis, and characteristics of prenatal counseling (Dorf et al. 2025). Parent‐related factors, including age, reproductive history, sociodemographic characteristics, and expectations regarding the child's prognosis and quality of life, were also examined (Dorf et al. 2025). While these studies provide insight into patterns and associations, they offer limited understanding of how parents interpret and make sense of the diagnosis during the decision‐making process.

Although a broader literature has examined aspects of parental experiences of receiving prenatal diagnoses involving variations of sex characteristics and other sex chromosome aneuploidies, including communication, information needs, and support (Whitehead et al. 2022; Jaramillo et al. 2019; Zampini et al. 2021), qualitative research on parental decision‐making in the specific context of TS remains scarce. Only two interview‐based studies have explored parental decision‐making following prenatal diagnosis of SCAs more broadly, and neither focused specifically on TS (Pieters et al. 2011; Balkan et al. 2010). These studies highlighted important considerations in parental decision‐making, including perceived severity of the conditions, moral or religious orientations, and engagement with information and counseling (Pieters et al. 2011; Balkan et al. 2010). However, they did not examine in depth how such considerations are interpreted, negotiated, and integrated over time and how they inform one another in the course of decision‐making. A deeper and more nuanced understanding of parental perspectives is important for the continued development of parent‐centered prenatal genetic counseling.

The aim of this exploratory qualitative study was therefore to examine how parents who continued a pregnancy following a prenatal diagnosis of TS experienced and navigated the decision‐making process, with the broader goal of informing clinically and psychosocially responsive prenatal counseling.

## 2 Methodology

### 2.1 Study Design

This qualitative interview study is grounded in a constructivist epistemological stance. From this perspective, parents' accounts are viewed as situated interpretations shaped by lived experience and context. Accordingly, experience is produced and articulated through language, and analysis is conceptualized as an interpretive process rather than as the identification of pre‐existing meanings.

### 2.2 Positionality Statement

The research team comprised five physicians and one anthropologist. The physicians included a clinical geneticist specializing in sex chromosome conditions, two pediatricians with expertise in TS, a physician training in clinical genetics, and a physician training in pediatrics. Collectively, they brought extensive experience in the diagnosis, treatment, counseling, and research of TS. The anthropologist contributed substantial expertise in qualitative health research, with a particular focus on prenatal diagnostics. All members of the research team are of Danish ethnic background and based in Denmark. Through their clinical and research practice, the team is committed to improving understanding, treatment, and the broader conditions of life for children with TS and their families. The interviewer had no prior relationship with the participants, although some members of the pediatric research team knew individual families through clinical care.

### 2.3 Study Setting

In Denmark, all pregnant people are offered publicly funded prenatal care, including combined first‐trimester screening and a routine second‐trimester ultrasound examination to detect fetal anomalies (The Danish Health Authority 2020). In cases of high‐risk screening results and/or suspected fetal anomalies, invasive prenatal genetic testing is offered (The Danish Health Authority 2020).

When a prenatal diagnosis of TS is established, the result is communicated to the pregnant person or couple by an obstetrician or a specialist in fetal medicine. Prior to decision‐making regarding pregnancy continuation or termination, the pregnant person or couple is usually offered prenatal counseling by a clinical geneticist and/or by a pediatrician with experience in TS. In the Danish healthcare system, prenatal genetic counseling is primarily provided by physicians specializing or training in clinical genetics, who therefore fulfill counseling functions that in other healthcare systems may be undertaken partly or entirely by non‐physician genetic counselors.

At the time of the participants' pregnancies, termination of pregnancy on request was legally permitted up to 11 + 6 weeks of gestation (Folketinget 2025). Termination beyond this gestational age required approval from a regional termination board. From our clinical experience, termination following a prenatal diagnosis of TS would typically be approved until 22 + 6 weeks of gestation.

### 2.4 Participants and Recruitment

Eligible participants were Danish parents who had received a prenatal diagnosis of TS and had decided to continue the pregnancy. To ensure relevance to contemporary clinical practices and to capture experiences that remained salient in their everyday lives, inclusion was further limited to parents of a daughter with TS aged 0–6 years at the time of interview. The study focused exclusively on parents who decided to continue the pregnancy to enable an in‐depth exploration of decision‐making experiences within this specific group.

Participants were recruited through purposive sampling via pediatric departments across Denmark, the Danish Turner Syndrome Patient Association, the Danish Turner Syndrome Facebook group, and lectures organized for patients with TS and their families. Recruitment continued until sufficient information power (Malterud et al. 2016) was considered to have been achieved. Parents of 13 children expressed interest and received additional information about the study. Parents of 12 children agreed to participate, while one mother did not respond after initial contact.

Twelve semi‐structured interviews were conducted. Both parents participated in seven interviews, while only the mother participated in five. Fathers' non‐participation was attributed to time constraints or to the view that the mother could adequately convey their shared experience. All non‐participating fathers expressed confidence in their partner's ability to convey their joint perspective.

Participants represented diverse educational and occupational backgrounds, including both blue‐ and white‐collar professions, and two families included members with ethnic backgrounds other than Danish. Additional interview and participant characteristics are presented in Table 1.

**TABLE 1:** Participant and interview characteristics.

Characteristic	Value^a^
Total number of interviews	12
Interview length (minutes)	90 (61–105)
Interview participants	
Both parents	7
Mother only	5
Maternal age (years)	36.5 (29–43)
Paternal age (years)	37 (30–46)
Age of daughter with Turner syndrome (years)	3.5 (< 1–5)
Parents cohabiting	12
Parent reporting religious practice	2
Fertility challenges and/or recurrent pregnancy loss	7
Number of children prior to the child with Turner syndrome	
0	4
≥ 1	8
Indication for invasive prenatal genetic testing	
Increased nuchal translucency/increased first‐trimester risk	8
Second‐trimester ultrasound abnormalities	3
Other	1
Prenatal karyotype	
45,X	4
45,X/46,XX mosaic	3
Other TS karyotype	2
Unknown TS karyotype	3
Recruitment pathway	
Pediatric departments	5
Patient networks^b^	5
Patient lectures	2

^a^ Values are presented as median (range) or as the number of interviews in which the characteristic was reported.

^b^ The Danish Turner Syndrome Patient Association or the Danish Turner Syndrome Facebook group.

### 2.5 Dataset Generation

Interviews were conducted between September 2024 and October 2025, either face‐to‐face (*n* = 7) or via video call (*n* = 5). All interviews were conducted by Inger Lily Hjuler Dorf, a medical doctor trained in clinical genetics, under ongoing supervision from Stina Lou, an experienced qualitative researcher. The semi‐structured interviews were based on an interview guide informed by clinical expertise within the author group and prior research (Pieters et al. 2011; Lou, Lanther, et al. 2020; Lou et al. 2021, 2025; Jun et al. 2018). An overview of interview topics is presented in Table 2. Eleven interviews were conducted in Danish and one in English, according to participant preference. The interviews were audio‐recorded and transcribed verbatim by Inger Lily Hjuler Dorf. Quotations from the Danish interviews were translated into English with attention to preserving idiomatic meaning and tone. Data were stored securely on a protected server within the Central Denmark Region.

**TABLE 2:** Key domains and illustrative questions from the semi‐structured interview guide.

Interview topics	Illustrative interview questions
The diagnostic process	“Can you tell me how it was discovered that your fetus might have Turner syndrome?” “How did you feel during the period between having the sample taken (e.g., chorion villus sample or amniocentesis) and receiving the result?” “What did you talk about? Did you talk to others?”
Receiving the diagnosis	“Could you describe the situation in which you received the result of the chorionic villus sampling/amniocentesis?” (Impressions of the physician, thoughts and emotions related to the result and the consultation)
Genetic counseling	“Can you tell me about the consultation?” (Impressions, thoughts, emotions, anything surprising) “How was Turner syndrome described?” (Risks, possibilities)
The decision to continue	“What concerns or thoughts were most prominent during the decision‐making process?” “Could you try to describe what shaped your decision to continue the pregnancy?” “Where did you seek information and guidance during the decision‐making process?” (Family/friends, health care professionals, the internet, patient organization)

As part of reflexive practice, Inger Lily Hjuler Dorf engaged in ongoing discussions with the research team throughout data collection concerning both interview content and the interview process. Drawing on her background in clinical genetics, Inger Lily Hjuler Dorf became attentive to how counseling practices—such as summarizing, validating, or steering toward conclusions—might shape the interview interaction. While central to clinical work, these practices required conscious moderation in the interview context, where participants were approached as experts on their own experiences. Inger Lily Hjuler Dorf reflected on how prior clinical knowledge and assumptions at times limited further exploration, for example, by presuming shared understandings rather than inviting participants to elaborate. These reflections informed a gradual shift toward a more open, participant‐led interviewing approach and a clearer distinction between the roles of clinician and qualitative researcher.

### 2.6 Data Analysis

Interviews were analyzed using reflexive thematic analysis as outlined by Braun and Clarke (Braun and Clarke 2024). The analysis was primarily inductive. This approach offers a flexible analytic framework for developing themes that capture shared patterns of meaning, while also attending to variation, nuance, and contradiction (Braun and Clarke 2024, 2006, 2022). Themes are conceptualized as analytic constructions developed through sustained engagement with the material, and the researcher's interpretative role is explicitly acknowledged (Braun and Clarke 2024, 2006, 2022).

Analysis was approached as an iterative process, in which meanings were actively developed through engagement with the material. The analytical phases are described in detail in Table 3.

**TABLE 3:** Application of the phases of reflexive thematic analysis.

Analytical phase	Analytic activities and reflections
Familiarization	Inger Lily Hjuler Dorf transcribed all interviews verbatim and read the transcripts repeatedly while documenting initial, unstructured reflections and points of analytic interest. Early reflections were discussed within the research team to support reflexive awareness of how interpretations were shaped through engagement with the data and researcher perspectives
Coding	Inger Lily Hjuler Dorf undertook inductive coding of a subset of interviews to explore what appeared central to parents' accounts. Initial codes and reflections were discussed with Stina Lou. On this basis, a coding guide was developed as a reflexive analytic tool to support continued engagement with the material rather than as a fixed or exhaustive framework All interviews were coded in NVivo (Lumivero, Denver, CO, USA). Inger Lily Hjuler Dorf reviewed the coded material in its entirety and produced detailed analytic notes examining relationships between codes and their relevance to the overarching analytic focus
Developing analytic focus	Through iterative engagement with the material, a central analytic focus gradually took shape concerning how parents' decision‐making was oriented around perceptions of ‘seriousness’, shaped by multiple influences and evolving over time
Constructing and refining themes	This analytic focus informed the development of an initial narrative account of the decision‐making process, including provisional themes. These were discussed within the author group and further refined through critical dialogue, resulting in the final themes and subthemes presented in the Results section A thematic diagram was developed to visually represent the final themes and their relationships, illustrating the overall process described by the parents rather than a linear or chronological sequence (see Figure 1)

## 3 Results

The analysis showed how the decision to continue pregnancy following a prenatal diagnosis of TS unfolded as a gradual process rather than a single moment of choice. Across interviews, parents engaged in a continuous calibration of what TS “meant” and how serious it felt in the context of their lives. Their assessments were shaped by prior experiences and values, emerging attachment to the fetus, information and communication from healthcare professionals, and responses from social networks. These dynamics are reflected in the interrelated themes and subthemes presented below (see Figure 1).

**FIGURE 1:**
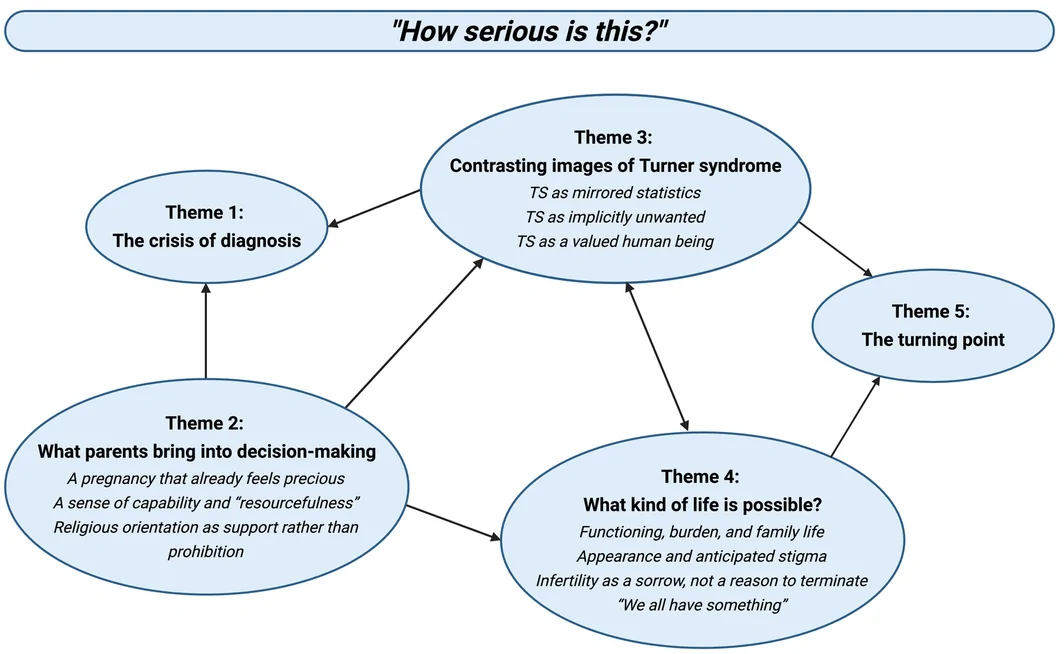
Thematic map. Created in BioRender. Skakkebæk, A. (2026) https://BioRender.com/4skh1xg.

### 3.1 Theme 1: The Crisis of Diagnosis

For most couples, the diagnostic process was described as a “life crisis.” The period of uncertainty—awaiting confirmatory results, additional testing, and serial ultrasounds—was experienced as emotionally and physically exhausting.

TS was unfamiliar to almost all parents, and the wide range of potential outcomes contributed to confusion; the diagnosis was described as difficult to “grasp,” both medically and existentially. One mother expressed:

‘What is Turner?’ I mean, that's kind of what you have to learn to live with. That you're given something you don't know what is, and there's no one who can tell you what it is. And that was what was hard during the pregnancy, that there was no one who could really tell us anything at all. (Mother 3)

This ambiguity was compounded by parents' simultaneous awareness of the ongoing risk of spontaneous pregnancy loss.

Differences in how parents navigated the situation added further strain for some couples. They described moving through the decision‐making process at different paces and with differing needs for information, discussion, and openness toward others. While many couples experienced these differences as manageable, others recalled having to work to regain trust and togetherness in the relationship:

It was very difficult for me, because at times you [husband] were leaning more toward not continuing – and I completely understand that. But for me, because the baby was growing inside my body, and because I became more and more attached, and because I think I convinced myself that I had some kind of feeling that this child was doing well, I felt very alone. And from my perspective, something happened between you and me during that process. We drifted further apart. We've somehow managed to make up for that since, but it was hard – because we weren't in the same place, and we didn't have the same need to talk it through. (Mother 7)

We had different approaches, right? You were very much about facts – reading and researching – and you couldn't really understand that I didn't have the same need to keep gathering more information. (Father 7)

For some parents, the sense of crisis did not resolve with the decision itself but persisted into late pregnancy or even beyond birth. A few mothers expressed retrospective moral distress, describing feelings of guilt or shock at having considered terminating a child they now loved:

My husband says that, even today, he still feels terribly guilty about even considering termination – and I do too, in some way. It's something that stays with you. That we could even think like that – that we might not want her. And that's just not how we feel at all today. It's a deep‐felt guilt, I think. And my husband says, ‘I wish I could forget all those thoughts I had. Because how am I ever supposed to explain that to her?’ And I keep trying to remind myself that we did choose to have her. (Mother 3)

In some cases, the mere existence of the choice—and the responsibility it entailed—became an enduring part of the crisis:

Having to make this decision also brought a lot of grief with it, because we were really close to terminating the pregnancy – it was almost fifty‐fifty. And the fact that we were even given that choice, and had to go through that whole process, stayed with us throughout the rest of the pregnancy. I remember before we made our decision, we were walking in a park, and our oldest daughter was sleeping in the stroller. And we talked about how, even if we had ended the pregnancy, there would always be a void [sobs] – where she should have been. (Mother 6)

At the same time, some parents described the eventual TS diagnosis as comparatively reassuring, as they had initially been warned about the possibility of more severe syndromes involving major malformations or intellectual disability. While this realization did not eliminate the crisis, it could mitigate the initial sense of alarm.

### 3.2 Theme 2: What Parents Bring Into Decision‐Making

Parents do not enter the prenatal diagnostic process as blank slates, but draw on life experiences, personal dispositions, and assumptions about parenthood and “the good life.” While these orientations were seldom explicitly articulated, several recurring patterns became evident in the analysis.

#### 3.2.1 A Pregnancy That Already Feels Precious

More than half of the interviewed couples described fertility challenges or recurrent pregnancy loss. For these parents, the pregnancy was often experienced as particularly precious even prior to the prenatal diagnosis of TS—sometimes explicitly framed as “a rare or final opportunity to have a child.” This context appeared to intensify the emotional stakes of contemplating termination, which was described not only as a decision about *this* fetus but also about the possibility of never having another child. One mother recalled:

And I remember saying to my husband, ‘I can't end it without knowing more. I must turn over every stone.’ Because this was once‐in‐a‐lifetime. We wouldn't get another chance, unlike the other 98% who choose to opt out and maybe can – because they're younger and because it's easier for them to get pregnant. (Mother 4)

Reproductive history also shaped early attachment. Mothers generally described developing a sense of attachment early in pregnancy, emphasizing physical and bodily connectedness as formative in their developing sense of motherhood. Several recalled a strong urge to protect the fetus, and for some, the diagnostic process felt like a threat to something hard‐won:

I was carrying her, and I was scared of… I was really scared to go to work. I was scared of jumping – afraid that something would go wrong. I didn't want to go to the swimming pool, because imagine if I got an infection. So, I developed this fear of losing her, because now she was finally there. And then the fear just… kept growing. (Mother 11)

Fathers, in contrast, more often described the fetus as abstract or distant for longer, with attachment developing more gradually:

I mean, I think it's hard to be completely objective about it, because as a man, you don't actually feel the pregnancy. I am a bystander. So, I don't form the same kind of bonds or feelings. (Father 7)

#### 3.2.2 A Sense of Capability and “Resourcefulness”

Many parents positioned themselves—explicitly or implicitly—as capable of meeting the demands associated with raising a child with potential health‐related or developmental challenges. Some couples recalled having discussed hypothetical scenarios even before receiving the TS diagnosis, often referring to conditions commonly framed as “more severe.” Several mothers described a prior orientation toward continuing the pregnancy even in such scenarios, grounded in a belief that they could still provide a good life for their child:

I think we were pretty open to the idea that even if it was Down syndrome, we would want to keep the child. So, I don't think we were ever in a place of, like, ‘Oh no, then we're not having this child.’ I think it was more like, ‘Okay… this just isn't going to be exactly how we expected.’ (Mother 10)

This orientation was closely tied to how parents understood themselves. Across interviews, parents described themselves as resilient, adaptable, or “better resourced than most,” emphasizing their perceived capacity to take on the practical and emotional demands associated with the diagnosis:

We have very deliberately decided that unless there's something lethally wrong, we will embrace the children we bring into the world. That's kind of what we've agreed on. And I believe that we'd be able to handle it. But I do know that we're relatively well‐resourced, and that others might find it really difficult to keep up with hospital appointments and having to give medication every day if that was necessary. So, parents are different and have different resources. I'm very aware of that. (Mother 3)

This self‐understanding appeared to support an interpretation of continuation as demanding, yet manageable within what they considered themselves able and willing to undertake.

#### 3.2.3 Religious Orientation as Support Rather Than Prohibition

In two interviews, parents described themselves as devout Christians. In these accounts, termination for fetal anomaly was not described as categorically prohibited, and parents did not feel constrained by religious rules. However, religious beliefs still shaped the emotional and moral texture of the decision, as ideas about the sanctity and worth of life made termination feel weightier.

Faith also provided support, offering meaning, community, and a hope‐oriented framework in which “miracles” were considered possible. As one mother described:

We prayed a lot. And I think the prayers kept us going. We also had friends from church who visited us. Like, they would come and sing for us and pray with us. And tell us positive stories: ‘You know, God can do things. He can change things around, you know?’ They talked about miracles happening. One lady was told, ‘Your kid will never walk’ – and she's walking. And at that time, we really needed that support a lot. I think that also kept us moving. At least we had hope, you know. (Mother 9)

References to miracles did not reflect an expectation that TS would be cured or disappear, but rather a belief that God might intervene to support the child and their family.

### 3.3 Theme 3: Contrasting Images of Turner Syndrome

The analysis revealed that parents actively sought information from multiple sources—including healthcare professionals, online materials, patient organizations, and their social networks—to form their understanding of TS. Analytically, this process could be understood as the negotiation of different representations of TS that shaped and reshaped parental understandings over time, depending on which aspects were foregrounded and how information was framed and interpreted.

#### 3.3.1 TS as Mirrored Statistics

All parents described statistical information as a central element in their evaluation of TS. Many recalled counseling consultations dominated by probabilities, risk estimates, and lists of potential complications. In some accounts, this included references to high termination rates following the prenatal diagnosis of TS, which introduced doubt and prompted reflection on whether important aspects of the condition might be overlooked:

There was also this feeling the doctor gave us, when she said, ‘You have a week to decide, and 98% choose to end the pregnancy.’ She said that, right? And already with those words, you start thinking, ‘Okay, there's something seriously wrong with my baby.’ You immediately imagine the worst. (Mother 4)

Rather than offering orientation, such presentations often left parents uncertain about how to interpret the diagnosis beyond numerical risk, particularly in the context of having received a diagnosis framed in terms of low probability:

But what I remember most from that conversation [with the clinical geneticist] is really feeling like they weren't telling me anything useful. It was very much like, ‘What's the risk?’ And lots of statistics. I just couldn't use that at all. Because if you look at the risk of having a child with Turner syndrome, it's almost non‐existent. But here I was, with a placental biopsy showing Turner syndrome, so that statistical information is completely useless to me. What I want to know is, ‘How are those Turner girls actually doing?’ And the geneticist wouldn't tell me that. It all became very numbers‐based, and I really didn't feel seen in that conversation. (Mother 3)

In contrast, some parents described encounters in which clinicians helped contextualize statistical information. By emphasizing uncertainty and vulnerability as inherent to all pregnancies, or by prioritizing the complications most relevant to the individual situation, statistical information was translated into a form that supported reflection rather than overwhelm:

[The geneticist] was very much like, ‘The important things to know are these: she won't be very tall, there's something about fertility, there could be something with the heart – but those are the things we can say. We can't say very much about everything else, because it's about statistics. These are the things you have to consider, and just like with your other children, they could run into all sorts of things later on.’ That was such a caring conversation. (Mother 1)

In the context of genetic counseling, parents generally did not find detailed explanations of genetics and chromosomal mechanisms meaningful. Parents often described such information as abstract and poorly aligned with the questions they were trying to answer at that stage, which centered on prognosis, quality of life, and possible futures for their child:

It was very factual. She was just drawing chromosomes, circling some of them, like, ‘She has these and not those.’ But I think, in our situation, we needed a different kind of information, right? More about what it actually meant for us. (Father 8)

#### 3.3.2 TS as Implicitly Unwanted

Parents' developing image of TS was also shaped by language, implicit messages, and social responses that positioned the diagnosis as undesirable. Certain words were described as emotionally charged and influential in shaping early perception. The term “syndrome,” in particular, was associated with Down syndrome and intellectual disability, while descriptors such as “disease,” “error,” or “deviation” were experienced as reinforcing fear and stigma:

When you haven't encountered a syndrome like this before, hearing the word ‘syndrome’ makes you think of Down syndrome. And I also think that the rhetoric around prenatal diagnostics is always about ‘risk,’ ‘error,’ and ‘disease.’ Why don't we say ‘variation’ or ‘difference’ instead? (Mother 3)

Even when parents later acquired more nuanced information, the emotional imprint of these terms sometimes persisted.

Clinical communication also conveyed implicit messages. Some parents described repeated references to termination as a legitimate and acceptable option, framed as both medically and morally permissible:

It was mentioned a lot: ‘You can also opt out,’ and ‘We can terminate,’ and ‘You still have time to terminate,’ and ‘You can terminate before a certain number of weeks…’ I mean, I remember that at some point along the way – whether it was around the 20‐week scan or something like that, I don't know – we were also told that you can terminate late. And then we were like, ‘Yeah, well, that's not what we're going to do.’ (Mother 12)

Such presentations fostered an understanding of TS as a condition one might reasonably opt out of.

Reactions from friends and family sometimes reinforced this perception. Some parents encountered pessimistic responses or concerns about the child's future or the parents' capacity to cope. Rather than changing parents' own appraisal, these responses often led to emotional distancing from family or friends, and, in some cases, strengthened parents' resolve to rely on their own values and judgments:

My mom was immediately like, ‘You don't want to go down that road. Then you're tied down for the rest of your life.’ I got more and more angry with her because she kept interfering and trying to steer my way of thinking. I really distanced myself from her during that period and didn't talk to her for a long time. (Mother 2)

#### 3.3.3 TS as a Valued Human Being

Alongside these representations, parents also described encounters that supported a more positive image of TS as compatible with a meaningful and valuable life. Counseling consultations that emphasized normal intelligence, available treatments, and the prospect of a good quality of life were perceived as particularly helpful in supporting a more grounded understanding:

My husband had a lot of questions, so the pediatrician made sure they were answered. He asked, ‘Do these kids go to special schools?’ and the pediatrician said, ‘No, these kids have normal IQ. They go to a normal school. Of course, they can struggle with math – but who doesn't struggle with math?’ Then my husband said, ‘Oh, they might be short,’ and then the pediatrician said, ‘She's a girl anyway. She doesn't have to be tall. And of course, there is growth hormone that can make her taller – or she could wear heels.’ [laughs] And my husband asked, ‘Oh, also, the baby can have a webbed neck. Like, what do we do about it?’ And the pediatrician said they can do, like, cosmetic surgery, so that one could be corrected as well. And he also said, ‘We check these kids regularly, and if there is anything, we can see it early. If there are, like, heart issues, they can be corrected.’ So, most of the things had solutions. (Mother 9)

Parents particularly valued healthcare professionals with clinical experience caring for girls and women living with TS. Knowing that clinicians could draw on insights beyond the diagnostic label and speak to how TS unfolds in everyday life contributed to a sense that their child was being understood as a person rather than as a prognosis:

I was called by one of the senior consultants who works with Turner syndrome. And there, I really felt taken by the hand. He explained things in much more plain language and told me that what I had read online can feel very overwhelming, because it lists everything that can happen – but that doesn't mean it will happen. The girls he sees in his clinic are just completely normal little girls, just a bit shorter, needing help to go into puberty and have children later on. And I think that's why it felt so good to talk to him. (Mother 5)

Reassurance that there was ample time to decide was commonly interpreted as an implicit signal that immediate termination was neither medically nor morally imperative, allowing parents to approach the decision with greater confidence and deliberation. One mother reflected:

[The doctor] didn't explicitly support us in one direction or another, but afterward we talked about whether there was still something in the way she kept saying, ‘You have plenty of time.’ I mean, it didn't come across as if she saw this as some completely insane syndrome where we had to rush to have an abortion. On the contrary, she was very much like, ‘Think it through, take your time, seek information.’ And although I know that you doctors aren't supposed to give your opinion, we later wondered whether she might also have thought that we should continue the pregnancy. But I don't feel that it influenced us in a negative way – more that it helped normalize it. (Mother 6)

Encounters outside the clinical setting also played an important role. Several parents sought contact with patient organizations or online peer communities, where they encountered others living with TS or parenting children with the condition. These encounters were described as emotionally significant, particularly when parents were exposed to images and narratives of children and families who were doing well. Being met with recognition and affirmation in these contexts contributed to a sense of relief and normalization:

And then I found the Turner syndrome Facebook group. And I posted something like, ‘Is there anyone else who has…? What are your experiences? Or can I see…?’ And I looked at pictures of the children in the group, or things like that. Yeah, I got a lot of good out of that [laughs]. And people were writing, ‘Oh, congratulations.’ Hardly anyone had congratulated me on the pregnancy before I went in there [cries]. Like, ‘Congratulations, you have a child with Turner syndrome.’ [laughs] It was kind of… it gave me a bit of peace. There were actually other people in this world who had children with Turner syndrome and who were doing well. (Mother 2)

Responses from family members and friends further situated the diagnosis within everyday life. Expressions of acceptance—such as relatives emphasizing that the child would be welcomed regardless of the diagnosis—helped some parents view continuation as both personally meaningful and socially possible:

My brother‐in‐law said, ‘But she is just going to be part of our big bunch – our big family.’ That meant so much to me: that others would just accept her as the child she is and not see her as something odd. (Mother 1)

Others valued a more restrained response, describing a need for others to listen rather than guide or influence the decision. In these contexts, TS was framed less as a defining limitation and more as one aspect of a child whose life was expected to hold inherent worth.

### 3.4 Theme 4: What Kind of Life Is Possible?

Parents' appraisals of TS often crystallized around life‐defining questions that extended beyond medical outcomes and reflected implicit understandings of what constitutes a good, dignified, and socially viable life.

#### 3.4.1 Functioning, Burden, and Family Life

The analysis revealed how assessments of seriousness were closely tied to expectations about everyday functioning, future independence, and the possibility of a life that could unfold meaningfully within a family context. Cognitive development was a central concern. Parents described the likelihood of normal intelligence as particularly important, as it was linked to education, employment, independent living, and the possibility of “an equal relationship” with their child over time:

We were so happy to realize that we could have an equal relationship with our daughter, even though she has Turner syndrome. That we were going to have a child with a language and all the things we were actually looking forward to. That it's not a syndrome that means you can't build a relationship with her or that she would have a completely different life from any potential siblings. (Mother 10)

In this sense, parents were not only imagining a child, but also an adult future and a place in society.

These reflections were interwoven with concerns about medical burden and care needs. Parents considered whether their daughter's life might involve frequent hospitalization or ongoing medical interventions, and what this would mean for the child's well‐being as well as for family routines, parental capacity, and siblings:

The main thing was obviously the child's well‐being, but at the same time, it couldn't come at the expense of her brother. If we would be running around to all kinds of appointments, all kinds of surgeries, and things like that, we felt that would affect him [older brother]. And we didn't think that would be fair to him. (Father 11)

Structural anomalies detected on ultrasound were therefore described as especially significant, as they were interpreted as indicators of more severe limitations and extensive medicalization. Several parents described postponing a final decision until the second‐trimester ultrasound, which functioned as a key point of orientation in evaluating both the child's prospects and the anticipated balance between medical care and ordinary life:

We were scanned regularly at the hospital to check the internal organs. We had talked a lot about it – that if there was something serious with the internal organs, then we would end the pregnancy. (Mother 8)

That's where we drew the line, so to speak. If there had been something seriously wrong with the organs – something that would affect her ability to have a normal life – that would probably have been a deal breaker. (Father 8)

#### 3.4.2 Appearance and Anticipated Stigma

Concerns about whether the child might “look different” elicited more divergent responses. Some parents described appearance as largely irrelevant or manageable, while others worried about stigma and negative social reactions:

I was a bit worried about, ‘What will she look like?’ Not because… I would love her unconditionally. I didn't have those feelings of, ‘Can I love a child who looks different?’ It was more that thing of, ‘Oh, what will other people think – will they treat her differently?’ (Mother 11)

These reflections highlighted that seriousness was interpreted not only medically, but also socially, through the anticipated responses from others.

#### 3.4.3 Infertility as a Sorrow, Not a Reason to Terminate

Infertility associated with TS was another topic characterized by variation in how parents reflected on its significance. Some parents expressed hope and openness toward multiple possible life paths, referring to medical advances, egg donation, adoption, or the possibility that their daughter might not wish to have children. Others described infertility as a profound sorrow and articulated a moral paradox: deeply wanting a child while potentially having a child who might not share that opportunity. Despite these differences, infertility was not described as a sufficient reason to terminate the pregnancy:

Yes, I think we did consider the risk that she might not be able to have children. We talked quite a lot about whether our own need to have a child could end up being so strong that we would have a child who, in principle, might not be able to have that same need fulfilled herself. But we also agreed that a lot can still happen in research, and that it shouldn't be the reason you end a pregnancy. (Father 7)

Notably, parents who had undergone fertility treatment did not appear to be more concerned about their daughter's potential infertility; instead, some described their own experiences as evidence of possibility rather than limitation.

#### 3.4.4 “We All Have Something”

When discussing learning difficulties or other comorbidities, many parents drew on a normalizing frame, emphasizing that all families live with challenges, and that risk alone did not justify termination. For some, family experiences with ADHD, autism, or dyslexia reinforced an understanding of difference as part of ordinary life rather than something that necessarily required a diagnostic label:

In my view, you could just as well be born with a leg that – well, like your friend, where they had a child, whose leg was turned the wrong way completely, right? I mean, that's not… that's very… that's something you can see. But like you say, there are also people who have ADHD, or people who have other things. Everyone is born with something. There's almost no one these days who doesn't have something wrong with them [laughs]. So that's just how we are as humans: we all have all kinds of things wrong with us. (Mother 2)

### 3.5 Theme 5: The Turning Point

Although the decision to continue the pregnancy was preceded by an extended period of uncertainty, information seeking, and deliberation, parents often described the moment of decision itself as distinctly bounded and decisive. After weeks of weighing risks, imagining futures, and revisiting the same questions, the decision felt not tentative but settled. In several accounts, this decisiveness was described against the backdrop of a longer process in which decision authority had already been negotiated. Mothers were often perceived as having the strongest claim to the final decision because they carried the pregnancy, a view that many fathers described as legitimate and natural:

It was a joint decision, but I wasn't the one who had to go through the physical part of it. So, we reached a shared decision, but I really think that her view probably carried a bit more weight. Because I wasn't the one who had to go through the physical side. (Father 1)

For some parents, a normal second‐trimester ultrasound precipitated the turning point, allowing deliberation to give way to commitment. For others, the decision crystallized during a specific clinical encounter in which existing information was brought together in a way that made continuation feel coherent and viable:

That conversation we had with [the pediatrician] changed everything for us. When we left, I felt like there wasn't a single question I could come up with that she hadn't already answered. She was so thorough in her explanations, and the way she put things into words really demystified everything about girls with Turner syndrome. She turned it into something normal, you could say. To paint the picture: We walk in, and there's just chaos in our heads. And when we leave, it's just – [exhales deeply] – our shoulders are all the way down, and we feel completely relieved, and it's just as if that cloud, that fog that was inside our heads, is just… gone. There's only blue sky. (Mother 4)

A few mothers described a less concrete but equally decisive shift, articulated as a strong sense that this particular child was meant to come into the world:

It was kind of there – when they got to those scans – that I became 100% convinced. Because that – together with my feeling that this was a child that was meant to be born – made me settle more into the pregnancy. (Mother 7)

While fear and concern did not disappear after the decision was made, parents expressed a clear change in orientation: the question of whether to continue the pregnancy was no longer revisited, and attention turned toward navigating the remainder of the pregnancy and preparing for the child's arrival:

Now that we've made the decision, I choose to believe that it will all turn out fine – and that hopefully a healthy girl will be born. (Father 7)

## 4 Discussion

The present study shows that decision‐making following a prenatal diagnosis of TS was a complex and emotionally demanding process. Parents experienced the diagnosis as a crisis, marked by urgency and uncertainty, and described actively working to form an understanding of TS that could support their decision.

These efforts unfolded in close interaction with multiple sources and were grounded in prior life experiences and personal values. Encounters within the healthcare system played a central role, particularly when clinicians translated medical knowledge into contextualized risk information that felt relevant and comprehensible. In contrast, highly technical accounts were often less meaningful. Responses from partners, family members, and peer communities contributed to how parents imagined their child's place within both their immediate family and the wider social world. Through this ongoing interplay of personal experience, clinical encounters, and social responses, parents gradually developed a coherent understanding of TS that ultimately crystallized into the decision to continue the pregnancy.

Our findings are consistent with previous research showing that parental decision‐making following a prenatal diagnosis of TS is shaped by perceptions of severity, the presence of ultrasound anomalies, moral or religious orientations, and the framing of information in counseling (Pieters et al. 2011; Dorf et al. 2025; Balkan et al. 2010). The present study extends this knowledge by showing how these elements interact and gain significance within parents' lived experience. For instance, in our material, severe ultrasound findings emerged as major sources of concern, as they were understood to have immediate implications for the child's health and survival and thus directly shaped assessments of seriousness. Rather than functioning as isolated determinants, different elements were described by parents as gaining influence through interpretation and integration into a broader understanding of what the diagnosis would mean for their child and their family life. In this sense, our findings move beyond demonstrating *that* certain factors matter, toward understanding *how* and *why* they come to matter in practice. Notably, while fetal karyotype emerged as a factor associated with parental decisions in the reviewed literature (Dorf et al. 2025), it did not play a prominent role in parents' own assessments of seriousness in this study. This suggests that genetic information influences decision‐making primarily through the meaning attributed to it in counseling encounters, rather than through the technical details themselves. Similar findings have been reported in studies of uncertain prenatal findings, where parents tend to recall the significance conveyed rather than the specific genetic information (Walser et al. 2016; Lou, Lomborg, et al. 2020).

Qualitative studies of parental decision‐making following other prenatal diagnoses report findings that resonate with those presented here. In a Danish interview study of parents who continued a pregnancy after a prenatal diagnosis of Down syndrome, Lou et al. describe how couples moved from an initial crisis response toward understanding the fetus as “the child we were given,” and how they actively sought experiential knowledge outside the hospital setting to imagine everyday life with the condition (Lou, Lanther, et al. 2020). Similar to our findings, the decision was framed less as an active selection of a child with a diagnosis and more as a response to an already existing child, with couples valuing contextualized, experience‐based accounts over abstract statistical risk. In another qualitative study of parents receiving uncertain prenatal genetic results, Lou et al. show how couples struggled to translate probabilistic and ambiguous findings into meaningful expectations about their future child, and how uncertainty persisted beyond the diagnostic encounter, requiring ongoing interpretative work (Lou et al. 2021). Parents in our study further highlighted that probabilistic information became even less useful once an outcome they had understood as highly improbable—a diagnosis of TS—had already occurred. This underscores parents' need to understand TS not as an abstract set of risks, but as a lived condition with implications for their child's health, well‐being, and everyday life (Jaramillo et al. 2019; Horowitz et al. 2025a).

The parents' experiences of making decisions in the context of a universal screening program may be understood in relation to the “expressivist objection” to prenatal screening, which draws attention to how the availability of screening and selective termination may communicate a negative valuation of particular conditions and of the people living with them (Boardman and Thomas 2023). Although participants did not explicitly criticize prenatal screening, some described how the repeated presentation of termination as an available option—including references to termination rates—was not experienced as a neutral expansion of choice, but as introducing a moral dilemma and potentially signaling that TS was a condition one might reasonably seek to avoid. This parallels findings by Steffensen et al., whose study of parents of children with Down syndrome found that some associated prenatal screening with broader social understandings of the condition as severe and undesirable (Steffensen et al. 2024). These accounts do not establish that healthcare professionals intended to communicate such a valuation, or that counseling was directive or coercive, but illustrate how the framing of reproductive options and population‐level estimates may itself acquire normative meaning for parents. This underscores the importance of contextualizing numerical information and clarifying that it does not constitute a recommendation.

In our study, confirmation of TS brought relief to some parents because it replaced fears of more severe conditions with a diagnosis they perceived as less serious. Drawing on qualitative studies of parental perspectives on differences or variations of sex characteristics, we suggest that this diagnostic clarification may also have narrowed an initially open‐ended uncertainty and created a more concrete space in which parents could begin to imagine their child's possible future. Horowitz et al. describe how diagnostic clarity may support parental understanding and preparation, while also showing that parents prioritized practical implications, psychosocial well‐being, and quality of life over etiological certainty (Horowitz et al. 2025b). Whitehead et al. similarly found that parents actively sought condition‐specific information and support to understand what prenatal findings might mean in practice (Whitehead et al. 2022). This resonates with our participants' emphasis on clinicians who could discuss TS beyond diagnostic labels and communicate how the condition might unfold in lived experience.

Not all decisions, however, appeared to result from such interpretative work alone. Several studies have found that when biomedical knowledge offers limited prognostic guidance, parents may also rely on social, religious, emotional, and moral frameworks, as well as embodied “gut instinct” (Lou et al. 2025; Horowitz et al. 2025a; Rubel et al. 2017; Cornwall 2021; Reed and Berrier 2017). In our material, several mothers likewise described a strong internal certainty that this particular child should come into the world, even in the absence of definitive medical reassurance.

Across these studies, clinical information did not determine decisions on its own but acquired meaning through integration with parents' prior experiences, values, and relational contexts. Whereas uncertainty in studies of ambiguous genetic findings often derives from limited knowledge of a variant's clinical significance, uncertainty in the context of TS is more commonly related to phenotypic variability and to how risks and future trajectories are framed in counseling. Despite these differences, parents across diagnostic contexts appear to engage in similar reflective work as they consider what a diagnosis might mean for their child and family life.

### 4.1 Clinical Implications

Our findings underscore that prenatal counseling in TS involves more than the transmission of biomedical facts. While clinicians must provide accurate information about the diagnosis and its possible implications, this study highlights that the meaning of such information depends on how it is framed, contextualized, and related to parents' lives. As emphasized by Werner‐Lin et al., effective prenatal counseling requires balancing scientific communication with the relational and psychosocial work through which families integrate uncertainty into meaningful decision‐making (Werner‐Lin et al. 2016).

In TS, uncertainty is inherent, as phenotypic variability makes it impossible to fully anticipate how the condition will unfold. Information can outline possible trajectories, but it cannot determine what the diagnosis will mean for a particular child or family. Counseling should include balanced, individualized information about potential medical, neurocognitive, educational, and social outcomes, as well as the need for lifelong multidisciplinary health monitoring. Although karyotype–phenotype associations may inform discussions of possible outcomes, neither the karyotype nor the observed degree of mosaicism can reliably predict the phenotype of an individual child (Gravholt et al. 2023).

Several parents described how differing emphases across clinical consultations—and, in some cases, a perception that healthcare professionals had limited familiarity with TS—increased their sense of uncertainty and complicated their efforts to form a coherent understanding. These findings highlight two interrelated priorities for clinical practice. First, they underscore the need for healthcare professionals involved in prenatal counseling to receive training that enables them to recognize and respond to the psychological and relational dimensions of parental decision‐making and to identify when additional psychosocial expertise is needed. Second, they point to the potential value of coordinated, multidisciplinary counseling in which relevant specialties—such as clinical genetics, fetal medicine, pediatrics, and adult endocrinology—participate in joint consultations. While the composition of such a multidisciplinary team may vary according to local healthcare structures and available resources, it should include appropriate psychosocial and counseling expertise (Gravholt et al. 2024; Horowitz et al. 2025b; Gramc 2024). Bringing together complementary areas of TS‐specific expertise may reduce inconsistencies in how information is weighted and communicated across professionals and may support a more coherent and individualized counseling process. This approach may also facilitate a more integrated, life‐course‐oriented understanding of the diagnosis. Importantly, this form of counseling should be offered as an optional resource to all parents, while recognizing that some—particularly those who decide early to terminate the pregnancy—may experience this type of counseling as unnecessary or burdensome.

These efforts may support coherence; however, they cannot eliminate the fundamental indeterminacy of TS. In this context, the professional role extends beyond providing contextualized information to supporting parents in recognizing and navigating this inherent uncertainty.

Support needs may also extend beyond the immediate decision‐making period. Across several interviews, parents described persistent guilt or moral distress related to having considered termination, extending beyond the decision itself and, in some cases, beyond the birth of the child. Clinicians' awareness of these reactions may enable them to acknowledge and normalize such feelings, create opportunities for parents to process them, reduce the isolation they may otherwise experience, and facilitate access to psychosocial support where needed.

### 4.2 Strengths and Limitations

A key strength of this study lies in the interdisciplinary composition of the research group and the integration of clinical and qualitative expertise. The team combined extensive clinical experience in TS across genetic, prenatal, and pediatric care with established methodological expertise in qualitative health research. This combination enabled contextual insight and sustained reflexive engagement with the data throughout the analytic process. Interpretations were developed through ongoing dialogue within the group, allowing professional preconceptions to be critically examined rather than assumed.

The study is further strengthened by a heterogeneous sample with broad variation in geography, educational background, and family situation, including participants from ethnic minority backgrounds. This diversity enhances the analytic breadth of the findings by enabling exploration of shared patterns across differing life situations, while remaining attentive to variation in how parents made sense of the diagnosis and the decision‐making process.

As a qualitative study, the aim was not statistical generalization but to develop analytically grounded insights into how parents make sense of a prenatal TS diagnosis. The findings should therefore be understood as situated interpretations developed within a clearly described Danish healthcare context. In particular, the availability of publicly funded specialist care and long‐term follow‐up may have influenced how parents understood and evaluated the anticipated consequences of the diagnosis. Rather than claiming representativeness in a quantitative sense, we have sought transparency in recruitment, data generation, and analysis to enable readers to assess the relevance and transferability of the findings to other cultural and institutional settings. Although no qualitative study can encompass all perspectives, deliberate efforts were made to include families with diverse backgrounds and circumstances to capture variation in experience. Recruitment through patient networks and educational events may nevertheless have preferentially reached parents who were more engaged with TS‐related communities, and the experiences of less‐connected families may therefore be underrepresented. By comparison, recruitment through pediatric departments provided access to families attending broadly available routine follow‐up care, although participation still depended on parents' willingness, interest, and capacity to take part. Families experiencing the greatest burden related to the diagnosis or other circumstances in their lives may therefore be underrepresented.

### 4.3 Future Research

While this study sheds light on the decision‐making process of parents who continue a pregnancy, qualitative studies exploring how parents who choose to terminate a pregnancy in light of a TS diagnosis experience and navigate that decision are needed. Such research could clarify which forms of uncertainty, moral reasoning, or contextual influences become more salient when the decision leads to termination.

In addition, the present study suggests that the decision to continue pregnancy should not be understood as an endpoint, but as the beginning of new phases of uncertainty and adjustment. Future qualitative studies could therefore examine how parents experience the remainder of the pregnancy and early parenthood in the context of a known diagnosis. This would allow exploration of how initial expectations, hopes, and concerns evolve over time and how ongoing clinical follow‐up and everyday family life shape parental experience.

## 5 Conclusion

This study demonstrates that decisions to continue a pregnancy following a prenatal diagnosis of TS emerge through a complex interplay of medical information, personal values, experiences, and social context. Rather than constituting a single moment of choice, decision‐making unfolded over time as parents made sense of uncertainty and imagined possible futures. These findings highlight the importance of prenatal counseling that goes beyond information provision and supports the interpretative and emotional work involved in such decisions.

## Author Contributions

**Inger Lily Hjuler Dorf:** conceptualization, investigation, writing – original draft, methodology, writing – review and editing, resources, visualization, project administration, formal analysis. **Stina Lou:** conceptualization, writing – original draft, methodology, writing – review and editing, supervision, formal analysis. **Christine Manich Bech Baggesgaard:** resources, writing – review and editing. **Rune W. Naeraa:** resources, writing – review and editing. **Casper P. Hagen:** resources, writing – review and editing. **Anne Skakkebæk:** supervision, writing – review and editing, writing – original draft, conceptualization, methodology.

## Funding

Inger Lily Hjuler Dorf and Anne Skakkebæk were supported by the Novo Nordisk Foundation (NNF23OC0082718).

## Ethics Statement

The study was approved by the Research Ethics Committee of Aarhus University (Journal no. 2024–0736839) and registered with the Aarhus University Record of Research Projects (Journal no. 2022–0367531) in accordance with Danish data protection legislation.

## Consent

All participants received written and oral information about the study prior to participation and provided informed consent. Participation was voluntary, and consent could be withdrawn at any time without consequences for current or future care.

## Conflicts of Interest

The authors declare no conflicts of interest.

## Data Availability

The data underlying this study are not publicly available due to the sensitive nature of the interviews and the absence of participant consent for data sharing. Access to de‐identified excerpts may be considered upon reasonable request to the corresponding author, subject to appropriate ethical approvals, data protection safeguards, and institutional agreements.
